# Case reports in medical education: a platform for training medical students, residents, and fellows in scientific writing and critical thinking

**DOI:** 10.1186/s13256-016-0851-5

**Published:** 2016-04-06

**Authors:** Aleksandra G. Florek, Robert P. Dellavalle

**Affiliations:** 1 Department of Dermatology, Northwestern University Feinberg School of Medicine, Chicago, IL USA; 2 Section of Dermatology, Veteran Affairs Medical Center, Denver, CO USA

## Abstract

A case report is a detailed narrative that usually illustrates a diagnostic or therapeutic problem experienced by one or several patients. Case reports commonly serve as the first line of evidence for new interventions or they function as alarms that an issue exists with an already established therapy. Case reports are of minor importance in evidence-based medicine; however, they make meaningful contributions to both the knowledge and education of medical students, residents, and fellows. Case reports are written with the goal of sharing information for medical, scientific, or educational purposes. They often serve as medical or even undergraduate students’ first experience with medical writing and they provide a solid foundation for manuscript preparation and publication. In the last few decades, there has been an exponential increase in medical student research, specifically in the number of manuscripts published by medical students. It is important to foster this academic spirit among students by encouraging them to become involved in research. This editorial will focus on the value and educational benefits of writing case reports for medical students, university students, residents, and fellows.

## Introduction

The role of case reports is to provide descriptive information about a clinical patient scenario and to share this educational experience with the general medical and scientific community [1]. Furthermore, case reports foster an educational medium in disseminating new and rare diseases, thus increasing the knowledge of evaluation, diagnosis, treatment, and prognosis of diseases [2]. This editorial outlines why traditional case reports are not only valuable to the general medical community, but are also an integral part of medical education and clinical training. This editorial also hopes to inspire medical students, residents, and fellows of all disciplines to write and continue writing case reports.

## From grand rounds to journals: importance of case presentation

Case presentation has always been a time-honored and important tool of medical education and inpatient care, and has consisted of presenting challenging medical cases to an audience of medical students, residents, and attending physicians [3]. Grand rounds has a tradition in medicine dating back to the presentations of Sir William Osler, known by many as the father of modern medicine, at Johns Hopkins School of Medicine. Often, patients with disorders that are rare, difficult to treat, or challenging to diagnose are discussed during grand rounds. Grand rounds function as a teaching tool to increase medical knowledge and improve medical care. By participating in these events, medical students and residents enhance critical reasoning skills and learn new information as well as ways to avoid potential mistakes.

Similarly to grand rounds, case reports are a valuable tool for medical students and residents to recognize clinical questions as they arise in the daily clinical practice. Students are then permitted to formulate an answerable clinical question, and then to find current best evidence to answer this question by performing a thorough and effective literature search. During the search, students critically appraise the medical literature and choose the appropriate literature to support the case. By writing a case report or case series, students gain experience in literature search and medical writing. Also, students execute the steps of evidence-based medicine, which consists of formulating a clinical question, finding the best evidence, critically appraising the evidence, and applying the evidence to the patient. Even if the manuscript does not ultimately get published, the review of the literature required to support the case carries educational value. From evaluating a patient’s medical history, to performing a physical examination, to considering various differential diagnosis, selecting a treatment plan, and considering various side effects and outcomes of treatments—all these components of case report write-ups provide an educational benefit to medical students, residents, and fellows.

Clinical learning during medical school and residency is to a large degree case-based [4]. Case presentations are often found in textbooks, conferences, daily team rounds, or departmental grand rounds. Case reports or presentations are an excellent tool for sharing educational experience. Some of the important educational objectives that case reports introduce include enhancing awareness of rare disorders to facilitate diagnosis, clarifying new aspects on the etiology of a disorder, clarifying misunderstood treatment response, and describing how to avoid future mistakes [5].

## Preparation for an academic career

Furthermore, writing a case report can be an excellent preparation or exercise in a medical student’s career, often preparing one for a scientific career [4]. Students will be able to add the publications to their curriculum vitae for residency and fellowship applications. They will also be able to present some of the cases at local, national, and regional medical conferences, enabling them to meet peers and faculty in the area of their interest, thus enhancing their networking skills.

## Case reports as vehicles for documenting new knowledge: first-line of evidence of new or innovative treatment

By writing case reports, medical students can contribute to the first line of evidence for developing new therapies [6–8]. In one case, a woman with a long-standing history of psoriasis was treated with infliximab, a tumor necrosis alpha-antibody. By just after two weeks after the infliximab infusion, her psoriasis had significantly improved. This isolated case report began a new era of treatment for psoriasis [9]. Albrecht et al. analyzed case reports and case series from *The Lancet*, published from 1 January 1996 to 30 June 1997, and found that of the 103 reports published in this high-impact journal, 24 were followed by randomized controlled trials (RCTs) [6].

## First and major source for detecting rare adverse events of treatment

Case reports may serve as a source for detecting rare adverse events, at times leading to the removal of drugs from the market. Going back to the example of psoriasis, the phenomenon of psoriasis exacerbation with the use of interferon alpha was first described in a case report [10]. There are abundant examples of severe side effects of drugs that were detected *after* the drugs were approved by the US Food and Drug Administration. Furthermore, case reports help to identify side effects due to interaction between drugs, as well as side effects due to drugs being given to patients with renal failure.

## First report of a rare condition

When encountering a rare pathology in the dermatologic clinic, one of the frequent procedures is to perform a search for similar cases in any of the search engines. A vigorous literature search in itself can be a valuable task assigned to a medical student.

## Consensus-based clinical case reporting guidelines

In 2013, a group of researchers published a set of systematic reporting guidelines to guide authors in writing case reports. The group published a 13-item checklist that provides a framework to fulfill the need for precision, completeness, and transparency for published case reports [11]. Implementing these guidelines will help medical students or residents write case reports that met these these reporting standards, with the aim of focusing on individualized patient care.

### Rapidity of publication

Compared to extensive studies such as RCTs, case reports offer the possibility of a quick publication, especially for busy clinicians or students who do not have the time or means of creating any prospective studies or RCTs [12]. Furthermore, there are many online open-access journals that focus on publishing case reports and case series, and that offer rapid publication times.

## Conclusion

Given the challenging and diverse nature of medicine, many medical students or residents will come across a perplexing patient case. By offering to write up such a case, medical students will learn how to perform a literature review, communicate with various physicians in charge with the care of the patient, structure a manuscript, gain consent from the patient, collect data from various sources in the clinic, and how to submit and revise the manuscript. All of these skills are worth crafting early in the career of a medical student. Additionally, reporting single cases and series of cases is an important aspect of clinical research, especially for medical students, who often have limited funds for research and eagerness to contribute to medical science.
